# Homologous and heterologous desensitization of guanylyl cyclase-B signaling in GH3 somatolactotropes

**DOI:** 10.1007/s00441-013-1763-y

**Published:** 2013-12-20

**Authors:** Iain R. Thompson, Samantha M. Mirczuk, Lorna Smith, Andrew J. Lessey, Bigboy Simbi, Andrew Sunters, Gary F. Baxter, Victoria J. Lipscomb, Imelda M. McGonnell, Caroline P. Wheeler-Jones, Abir Mukherjee, Mark S. Roberson, Craig A. McArdle, Robert C. Fowkes

**Affiliations:** 1 Endocrine Signaling Group, Comparative Biomedical Sciences, Royal Veterinary College, University of London, Royal College Street, London, NW1 0TU UK; 2 Development & Reproduction Group, Comparative Biomedical Sciences, Royal Veterinary College, University of London, Royal College Street, London, NW1 0TU UK; 3 Vascular Cell Biology Group, Comparative Biomedical Sciences, Royal Veterinary College, University of London, Royal College Street, London, NW1 0TU UK; 4 Clinical Sciences & Services, Hawkshead Lane, North Mymms, Hatfield, Hertfordshire UK; 5 Division of Pharmacology, The Welsh School of Pharmacy, Cardiff University, King Edward VII Avenue, Cardiff, CF10 3NB UK; 6 Department of Biomedical Sciences, College of Veterinary Medicine, Cornell University, Ithaca, NY 14853 USA; 7 Laboratories for Integrative Neuroscience and Endocrinology, Department of Clinical Sciences at South Bristol, University of Bristol, Whitson Street, Bristol, BS1 3NY UK

**Keywords:** Natriuretic peptides, Guanylyl cyclases, cGMP signaling, Protein phosphatase, Protein kinase C

## Abstract

The guanylyl cyclases, GC-A and GC-B, are selective receptors for atrial and C-type natriuretic peptides (ANP and CNP, respectively). In the anterior pituitary, CNP and GC-B are major regulators of cGMP production in gonadotropes and yet mouse models of disrupted CNP and GC-B indicate a potential role in growth hormone secretion. In the current study, we investigate the molecular and pharmacological properties of the CNP/GC-B system in somatotrope lineage cells. Primary rat pituitary and GH3 somatolactotropes expressed functional GC-A and GC-B receptors that had similar EC_50_ properties in terms of cGMP production. Interestingly, GC-B signaling underwent rapid homologous desensitization in a protein phosphatase 2A (PP2A)-dependent manner. Chronic exposure to either CNP or ANP caused a significant down-regulation of both GC-A- and GC-B-dependent cGMP accumulation in a ligand-specific manner. However, this down-regulation was not accompanied by alterations in the sub-cellular localization of these receptors. Heterologous desensitization of GC-B signaling occurred in GH3 cells following exposure to either sphingosine-1-phosphate or thyrotrophin-releasing hormone (TRH). This heterologous desensitization was protein kinase C (PKC)-dependent, as pre-treatment with GF109203X prevented the effect of TRH on CNP/GC-B signaling. Collectively, these data indicate common and distinct properties of particulate guanylyl cyclase receptors in somatotropes and reveal that independent mechanisms of homologous and heterologous desensitization occur involving either PP2A or PKC. Guanylyl cyclase receptors thus represent potential novel therapeutic targets for treating growth-hormone-associated disorders.

## Introduction

C-type natriuretic peptide (CNP) is the third member of the natriuretic peptide family and is highly conserved between species as diverse as elasmobranchs and humans (Fowkes and McArdle 2000). The vast majority of CNP effects are mediated via the specific guanylyl cyclase-B (GC-B) receptor, encoded by the *Npr2* gene and lead to a localized increase in cyclic guanosine 3’,5’ monophosphate (cGMP) signaling (Potter et al. 2006). CNP and GC-B are expressed in numerous tissues, including the major endocrine glands and throughout the central nervous system (Fowkes and McArdle 2000; Potter et al. 2006). The anterior pituitary is a major site of CNP expression, where it is predominantly localized in gonadotropes (McArdle et al. 1993) and acts as a local mediator of gonadotrope function (McArdle et al. 1994; Thompson et al. 2009). Mouse models of disrupted CNP or GC-B (*Nppc* ^*−/−*^ and *Npr2* ^*−/−*^, respectively) essentially phenocopy one another, in that these animals suffer from severe achrondroplasia and dwarfism, female infertility and early death (Chusho et al. 2001; Tamura et al. 2004). In addition, global *Npr2* ^*−/−*^ knock-out mice are also growth-hormone-deficient, suggesting a pituitary phenotype (Tamura et al. 2004). We have recently described the presence of both *NPPC* and *NPR2* transcripts in normal human pituitary tissue of fetal and adult origin and in a range of pituitary adenomas including those from acromegalic patients (Thompson et al. 2012). Despite this, the major role for CNP in pituitary function remains unclear.

Early studies indicate that atrial natriuretic peptide (ANP; Inagaki et al. 1986) is expressed in the anterior pituitary and it has been reported to act as a gonadotropin secretagog (Horvath et al. 1986). However, specific preparations of ANP were subsequently revealed to have been contaminated with gonadotrophin-releasing hormone, which accounted for the apparent effects of ANP on the secretion of luteinizing hormone (Abou-Samra et al. 1987). Nevertheless, several pharmacological studies support a functional role of ANP, acting via GC-A receptors, in the anterior pituitary (McArdle et al. 1993; Thompson et al. 2009; Gilkes et al. 1992; Fowkes et al. 2000) but, in keeping with the current understanding of the pituitary CNP system, the biological consequence of ANP signaling in the pituitary is unclear. ANP probably performs an alternative role to CNP, as genetic disruption to either *Nppa* (encoding ANP) or *Npr1* (encoding the GC-A receptor) fails to recapitulate the apparent growth hormone deficiency or female infertility and early death (John et al. 1995; Lopez et al. 1995).

Pharmacological regulation of the GC-B receptor has been elegantly described through many studies by Potter and Hunter (1998a, 1999) who have shown that dephosphorylation of specific serine and threonine residues of the GC-B receptor leads to rapid homologous desensitization. In keeping with the extensive literature describing the regulation of GC-A receptors (Potter et al. 2006), enhancement of protein kinase C (PKC) activity leads to heterologous desensitization (Potter and Hunter 2000). Prolonged exposure to ANP or CNP does not appear to cause agonist-induced internalization (Fan et al. 2005; Dickey et al. 2011), although this remains a controversial issue (Pandey 2005). Whereas these comprehensive studies have elucidated mechanisms of GC-B desensitization, the vast majority of them characterize the function of exogenously expressed receptors (Potter et al. 2006). In contrast, we have previously shown that endogenous GC-B receptors undergo both homologous and heterologous desensitization in gonadotrope-derived αT3-1 cells (Fowkes et al. 2000) and have characterized an intact CNP/GC-B system within gonadotropes and human pituitary tissue (Thompson et al. 2009, 2012).

Despite studies suggesting an effect of CNP and GC-B signaling in pituitary somatotropes (Hartt et al. 1995; Shimekake et al. 1994), little is currently understood about the natriuretic peptide system in these cells. In our current study, we examine the molecular and functional characteristics of the natriuretic peptide system in GH3 somatolactotropes and primary rat pituitaries. We describe an intact CNP/GC-B system in both GH3 cells and rat pituitary tissue and provide evidence for the regulation of GC-B signaling that involves both homologous and heterologous desensitization of cGMP signaling, potentially via a PKC-dependent mechanism but not altered GC-B expression or agonist-induced internalization.

## Materials and methods

### Materials

Materials and pharmacologic inhibitors were purchased either from Calbiochem (MerckMillipore UK, Feltham, UK) or Sigma (Sigma-Aldrich, Dorset, UK). GC-A (PGCA-101AP), GC-B (PGCB-101AP) and β-actin (ab8226) primary antibodies were purchased from Fabgennix (Frisco, Tex., USA) or AbCam (Cambridge, UK). All secondary antibodies were from DAKO (Cambridge, UK).

### Cell culture

GH3 cells were maintained in high-glucose DMEM (Sigma), supplemented with 10 % (v/v) fetal calf serum (FCS; Sigma), 100 U penicillin/ml and 100 μg streptomycin sulfate/ml (Sigma) in 5 % CO_2_ humidified air at 37 °C, as described previously (Fowkes and Burrin 2003). These growth hormone and prolactin-secreting cells were originally derived from Wistar-Furth rats with pituitary tumors (Tashjian et al. 1968; Faivre-Bauman et al. 1975) and express functional thyrotrophin-releasing hormone (TRH) receptors (Hinkle and Tashjian 1973) but not growth-hormone-releasing hormone receptors (Zeytin et al. 1984). Consequently, they are considered a hybrid somatolactotrope-derived cell line.

### RNA extraction and polymerase chain reaction

Total RNA was extracted from 1 × 10^6^ GH3 cells or from male Sprague Dawley rat pituitaries by using Tri-reagent (Sigma-Aldrich, Poole, UK) and subjected to DNase treatment (Qiagen, Manchester, UK) before generation of first-strand cDNA (Applied Biosystems, Warrington, UK). Polymerase chain reaction (PCR) was performed for a range of targets for 30 cycles by using the primers and conditions published previously (Thompson et al. 2009). In some instances, GH3 cells were treated with 0 or 100 nM CNP for 24 h prior to RNA extraction. These samples were processed for qualitative PCR (qPCR) analyses with commercially available rat *Npr2* and *18S* primer sets (Qiagen, Manchester, UK) under standard SYBR conditions as described previously (Bayol et al. 2010).

### Immunoblotting

GH3 cell lysates (from 1 × 10^6^ cells) were prepared in lysis buffer (0.5 M TRIS pH 6.8, 10 % [v/v] glycerol, 1 % [w/v] SDS). Protein lysates were sonicated, boiled at 95 °C for 5 min, resolved by 10 % SDS-polyacrylamide gel electrophoresis, transferred to polyvivylidene difluoride membrane and probed for GC-B (1:2000 dilution) or β-actin (1:5000 dilution) expression. Appropriate horseradish-peroxidase-conjugated secondary antibodies were purchased from DAKO.

### cGMP enzyme-immunoassay to measure total cGMP accumulation

GH3 cells were plated and underwent the respective treatments in physiological saline solution (PSS; 127 mM NaCl, 1.8 mM CaCl_2_, 5 mM KCl, 2 mM MgCl_2_, 0.5 nM NaH_2_PO_4_, 5 mM NaHCO_3_, 10 mM glucose, 0.1 % BSA, 10 mM HEPES, adjusted to pH 7.4). All pre-treatments were performed in the absence of 3-isobutyl-1-methylxanthine (IBMX), whereas all stimulations were performed in the presence of 1 mM IBMX. Each reaction was terminated with the addition of ice-cold 100 % (v/v) ethanol and the samples were dried down under vacuum as described previously (Thompson et al. 2009). Reagents and standards were prepared by using the instructions supplied with the cGMP enzyme-immunoassay kit (R&D Systems, Abingdon, UK). The optical density of each sample at 450 nm was determined by means of an Berthold Technologies Mithras LB940 plate reader with MicroWin 4.40 associated software (Berthold Technologies, Harpenden, UK).

### Immunofluorescence cytochemistry

GH3 cells were mounted on poly-L-lysine-coated coverslips and treated with either 0 or 100nM ANP or CNP in PSS during the indicated time points. Following treatment, cells were fixed with 4 % (w/v) formaldehyde for 10 min at room temperature and permeabilized with buffer (1 % [v/v] Tween-20 in 50 ml phosphate-buffered saline [PBS]) before incubation for 10 min at room temperature. Cells were then blocked with 3 M sodium azide dissolved in 92 % PBS, 3 % FCS and 5 % Tween-20 (v/v) for 20 min at room temperature, washed, then exposed to 1:200 diluted primary antibody solution (GC-A or GC-B, plus 0.1 % FCS and 0.1 % Tween-20) for 16 h at 4 °C. After further washes and a 5-min PBS immersion at room temperature, secondary antibody was added plus phalloidin-rhodamine β-actin (Invitrogen, Paisley, UK) and 4,6-diamidino-2-phenylindole (DAPI) nuclear counterstains (Vector Laboratories, Peterborough, UK) and incubated for 45 min at room temperature. Following three PBS washes and a final 5-min PBS immersion at room temperature, the slides were mounted in Vectorshield (Vector Laboratories, Peterborough, UK) on standard VWR (VWR International, Leicestershire, UK) slides ready for analysis. A Leica SPS confocal scanning laser microscope was used to capture the staining, which was analyzed with Leica SPS software (Leica Microsystems, Milton Keynes, UK).

### Statistical analyses

All graphs were generated and statistical analyses were performed by using Prism software (Prism 5.0 for Mac OS X, Graphpad Software, Calif., USA). Unless otherwise stated, one-way analysis of variance followed by *post-hoc* Bonferroni Multiple Comparison Tests were carried out to determine statistical significance (at *P* < 0.05). Numerical data presented in this manuscript represent the means ± SEM from at least three independent experiments, each performed in triplicate.

## Results

### Molecular and pharmacological characterization of natriuretic peptide receptors in GH3 somatolactotropes and primary rat pituitary tissue

**Fig. 1:**
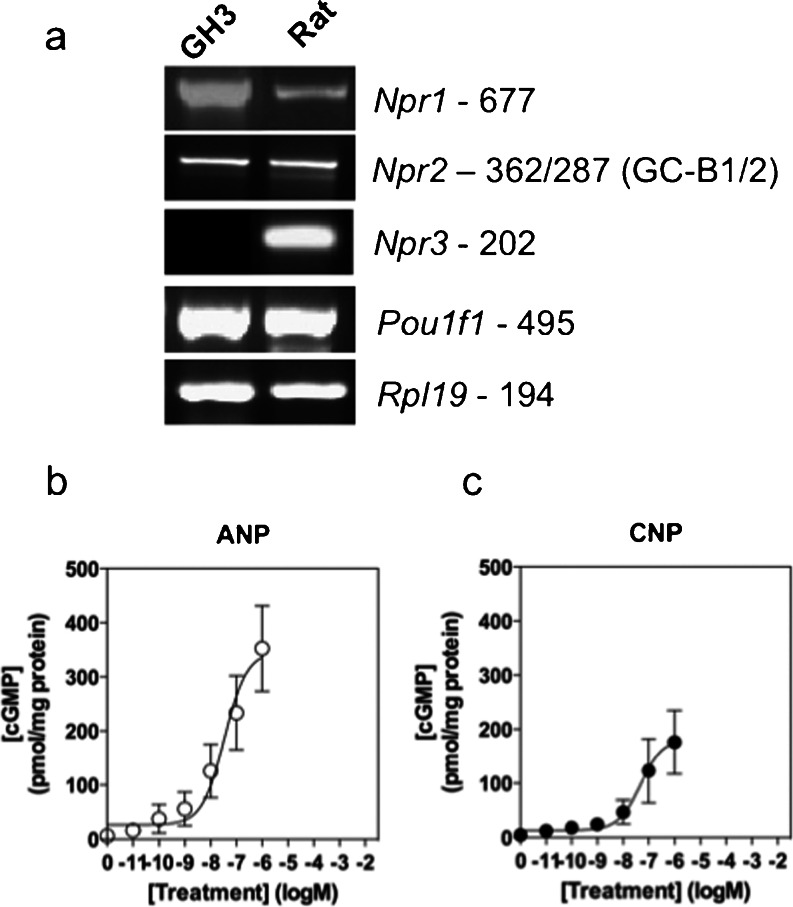
Molecular and pharmacological characterization of natriuretic peptide receptors in GH3 somatolactotropes and primary rat pituitary tissue. **a** Total RNA was extracted from either GH3 cells or primary rat pituitaries, prior to cDNA synthesis and reverse transcription plus the polymerase chain reaction for the indicated gene targets. Data shown are representative of at least three independent experiments. **b**, **c** GH3 cells were stimulated with the indicated concentrations of (**b**) atrial natriuretic peptide (*ANP*) or (**c**) C-type natriuretic peptide (*CNP*) in physiological saline solution (PSS) containing 1 mM 3-isobutyl-1-methylxanthine (IBMX) for 15 min, before termination with 100 %(v/v) ice-cold ethanol. Following extraction under vacuum, total cGMP accumulation was measured by enzyme-immunoassay and concentration-response curves were constructed by using pre-existing equations in GraphPad Prism 5.0. The data shown are means ± SEM of three independent experiments, each performed in triplicate and are expressed as pmol/mg protein (*n* = 3)

Our previous studies have revealed that *Npr1*, *Npr2*, *Npr3* and *Nppc* are all expressed in mouse pituitaries and/or in gonadotropes, suggesting a local role in the regulation of reproduction (Thompson et al. 2009). However, *Npr2* ^*−/−*^ not only are infertile but also have dwarfism and growth hormone deficiencies, suggesting that natriuretic peptides and their receptors also control somatotropes. To establish which guanylyl cyclases the GH3 somatolactotropes express, total RNA was extracted from GH3 cells and normal rat pituitary tissue, before qualitative reverse transcription (RT)-PCR. As shown (Fig. 1a), in GH3 cells, specific transcripts were detected for *Npr1* (GC-A), *Npr2* (GC-B1, GC-B2) and the somatotrope transcription factor, *Pou1f1* (Pit1). Interestingly, no expression of *Npr3* (clearance receptor) was detectable. In contrast, specific transcripts were detected for all these genes in primary rat pituitary tissue.

To establish whether functional GC-A and GC-B receptors were expressed in GH3 cells, we determined the effects of ANP and CNP on cGMP accumulation. GH3 cells were treated for 15 min with the indicated concentrations of natriuretic peptide, in the presence of 1 mM IBMX to inhibit phosphodiesterase activity. As shown (Fig. 1b, c), both ANP and CNP caused concentration-dependent increases in cGMP accumulation in GH3 cells (ANP EC_50_ ~ 45.7 nM, CNP EC_50_ ~ 53.7 nM).

### Evidence of homologous desensitization of endogenous GC-A- and GC-B-mediated signaling in GH3 cells

**Fig. 2:**
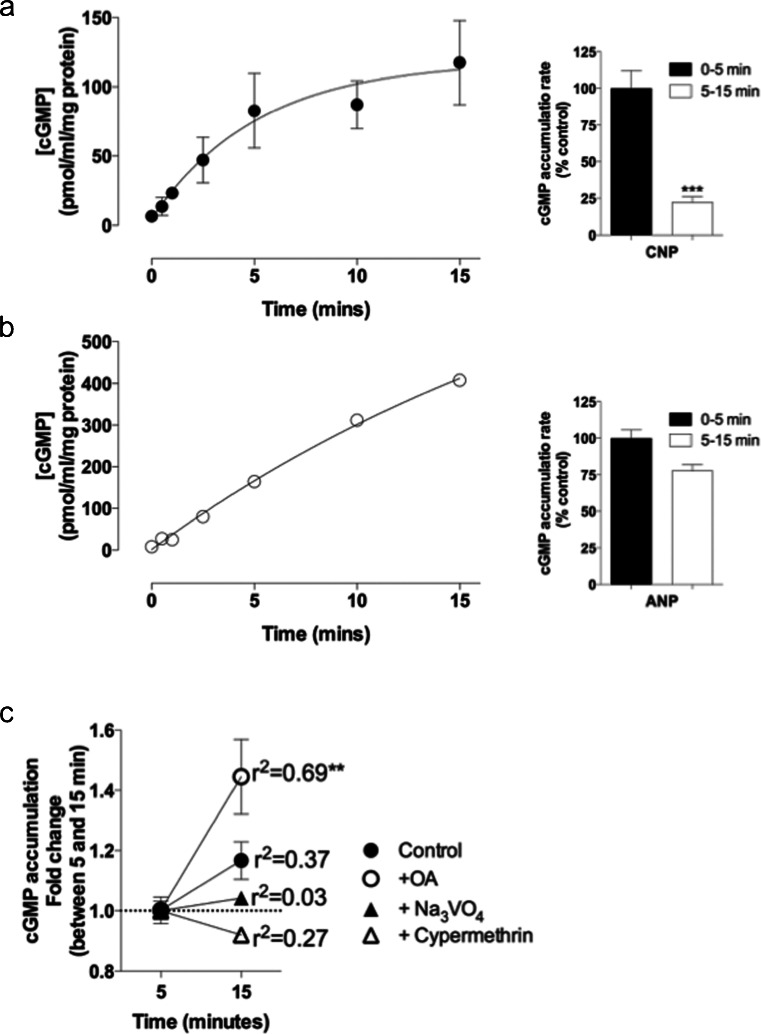
Rapid homologous desensitization of CNP-dependent but not ANP-dependent cGMP accumulation in GH3 cells. Cells were treated with PSS containing either (**a**) 100 nM CNP or (**b**) 100 nM ANP for 0–15 min in the presence of 1 mM IBMX. The data shown are means ± SEM of three independent experiments, each performed in triplicate and are expressed as pmol/ml per milligram protein (*n* = 3). The bar charts (*right*) indicate a comparison of the rate of cGMP accumulation between 0 and 5 min and between 5 and 15 min of stimulation (***P < 0.001, significantly different from the rate of cGMP accumulation between 0 and 5 min). **c** GH3 cells were treated as before with 100 nM CNP from 0 to 15 m in the presence or absence of either the protein phosphatase 2A (PP2A) inhibitor, okadaic acid (*OA*, 100 nM), the PP2B inhibitor, cypermethrin (1 nM), or the protein tyrosine phosphatase inhibitor, sodium orthovanadate (*Na*_*3*_ *VO*_*4*_, 1 mM) and assayed for total cGMP concentration. The data are means ± SEM of three independent experiments, each performed in triplicate and are expressed as the fold change in the rate of cGMP accumulation between 5 and 15 min (*n* = 3; **P < 0.01, cGMP accumulation maintained at a significant rate)

To characterize the activity of these receptors further , a series of experiments was performed to examine mechanisms of receptor desensitization. Total cGMP accumulation was measured to determine the changes in activity of the GC-A and GC-B receptors when treated over a short time-course with their native ligands, namely ANP and CNP, respectively. GH3 cells were plated at 3 × 10^5^ cells/well in a 24-well plate, treated with 100 nM of either ANP or CNP in the presence of the phosphodiesterase inhibitor IBMX (1 mM) for 0 to 15 min and assayed for total cGMP concentration (Fig. 2a, b). From 0–5 min of treatment with 100 nM CNP, a rapid increase in cGMP accumulation was seen from 6.4 ± 0.7 to 82.7 ± 15.5 pmol/ml per milligram protein. In contrast, cGMP accumulation between 5 to 15 min of treatment with 100 nM CNP (82.7 ± 15.5 to 117.4 ± 17.6pmol/ml per milligram protein) was significantly attenuated compared with that seen over the first 5 min (reduced to 22.2 ± 3.9 % of the initial rate of accumulation between 0 to 5 min; ***P < 0.001). This failure to maintain the initial rate of cGMP accumulation suggested that rapid homologous desensitization of GC-B-mediated cGMP signaling had occurred. In another experiment performed with 100 nM ANP instead of CNP, a rapid increase in cGMP accumulation (7.91 ± 0.9 to 164.31 ± 9.1pmol/ml per milligram protein) occurred between 0 and 5 min. In contrast to GC-B, this initial rate of cGMP accumulation was maintained for at least 15 min, suggesting that rapid homologous desensitization of GC-A-mediated cGMP signaling did not occur (Fig. 2b).

As previous studies have suggested that receptor dephosphorylation mediates desensitization of GC-B receptors (Potter 1998), the effect of inhibiting protein phosphatases was examined. As ANP failed to desensitize the GC-A receptor in these studies within 15 min, the effects of protein phosphatase (PP) inhibitors on GC-B were examined instead. Similar short time-courses were performed, except that some cells were pre-treated with 100 nM okadaic acid (OA, a PP2A inhibitor), 1 nM cypermethrin (a PP2B inhibitor), or 1 mM sodium orthovanadate (Na_3_VO_4_, a protein tyrosine phosphatase inhibitor) for 30 min prior to CNP treatment (Fig. 2c). As expected, control cell response to CNP underwent desensitization as shown by the failure to maintain the initial rate of cGMP accumulation. The initial rate of cGMP accumulation between 0 and 5 min was not significantly affected by any pre-treatments. Interestingly, cells pre-treated with OA maintained a significant rate of cGMP accumulation from 5 to 15 min (r^2^ = 0.69, **P < 0.01), whereas cypermethrin- and Na_3_VO_4_-treated cells failed to maintain a significant rate of cGMP accumulation, indicating a potential role for PP2A but not PP2B or tyrosine phosphatases, in mediating homologous desensitization of GC-B signaling in GH3 cells.

### Effect of pre-treatment on GC-A and GC-B receptor signaling in GH3 cells

**Fig. 3:**
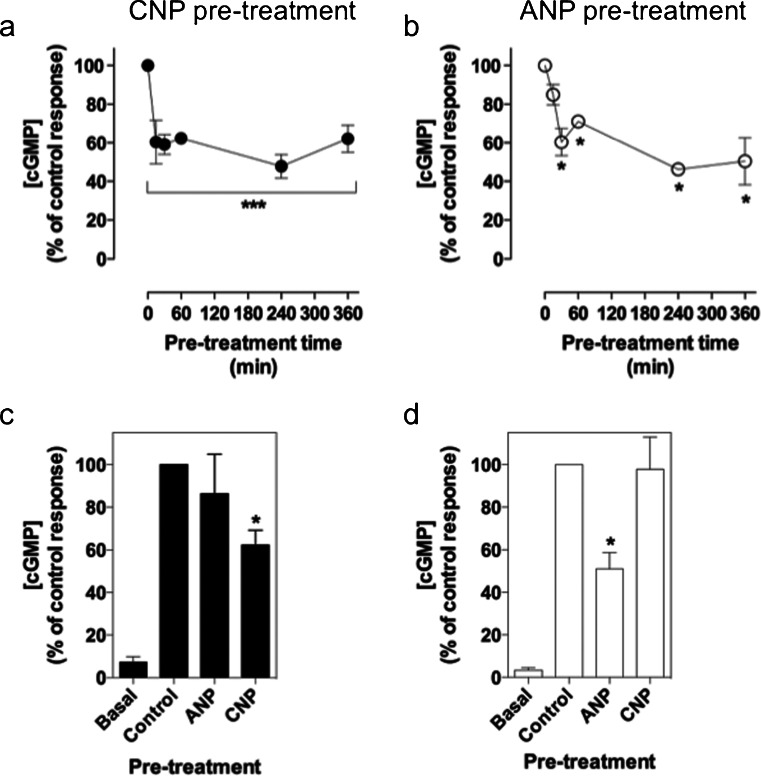
Effect of chronic exposure to CNP and ANP on GC-B- and GC-A-dependent cGMP accumulation in GH3 cells. Cells were initially treated with (**a**) 100 nM CNP or (**b**) 100 nM ANP in PSS without IBMX for 0 to 6 h. The medium was removed and replaced with 100 nM CNP (**a**) or 100 nM ANP (**b**) for 15 min in the presence of 1 mM IBMX. The data shown are means ± SEM of three independent experiments, each performed in triplicate and are expressed as the percentage of the control (*n* = 3). ***P < 0.001, *P < 0.05, significantly different from the control. **c**, **d** Cells were initially treated with 0, 100 nM CNP, or 100 nM ANP for 6 h as before. All pre-treatments were removed and replaced either with (**c**) 0 or 100 nM CNP or with (**d**) 0 or 100 nM ANP, for 15 min in the presence of 1 mM IBMX. The data shown are means ± SEM of three independent experiments, each performed in triplicate and are expressed as the percentage of the control (*n* = 3). *P < 0.05, significantly different from the control

Having established that GC-B signaling but not that of GC-A receptors, underwent rapid homologous desensitization, we next examined the effect of prolonged exposure of each receptor to its specific ligand. GH3 cells pre-treated with either ANP or CNP for 0 (control) to 6 h reduced the response to a subsequent 15-min treatment of ANP or CNP (with IBMX). GH3 cells pre-treated with 100 nM CNP demonstrated a significant reduction in cGMP produced compared with the control response to CNP (no CNP pre-treatment). The amount of cGMP measured from the 15-min pre-treatment sample was reduced by 39.6 ± 0.002 % (***P < 0.001). This effect was maintained up to the 6-h pre-treatment with CNP, which was reduced by 37.9 ± 7.1 % compared with the control (***P < 0.001). The reduction in cGMP accumulation compared with the control did not significantly differ between the 15-min to 6-h pre-treatments (not significant [ns], *P* > 0.05; Fig. 3a), suggesting that maximal desensitization of GC-B signaling occurred within 15 min of pre-treatment.

In similar experiments designed to examine the effect of ANP pre-exposure to GC-A activity, a significant reduction in cGMP accumulation was observed within 30 min of ANP pre-treatment (by 28.9 ± 3.1 % compared with the control, *P < 0.05). This inhibitory effect was maintained throughout the 6-h pre-treatment (*P < 0.05). Interestingly, GH3 cells pre-treated with ANP for 15 min did not show a significant reduction in cGMP accumulation compared with the control (ns, *P* > 0.05; Fig. 3b). In order to test the receptor specificity of the observed down-regulation of GC-B upon pre-treatment with CNP and of GC-A upon pre-treatment with ANP, the effects of ANP on GC-B and CNP on GC-A desensitization were determined. GH3 cells pre-treated with 100 nM CNP for 6 h caused a significant 37.9 ± 7.1 % (*P < 0.05) reduction in cGMP when subsequently treated with 100 nM CNP plus IBMX for 15 min, compared with the control. The amount of cGMP measured from the ANP pre-treated cells was not significantly different from the control samples (ns, *P* > 0.05; Fig. 3c). Pre-treating GH3 cells with 100 nM ANP for 6 h resulted in a significant 50.0 ± 7.7 % (*P < 0.05) reduction in cGMP produced when cells were then treated with 100 nM ANP plus IBMX for 15 min, compared with the control. The amount of cGMP measured from the CNP pre-treated cells was not significantly different from the control samples (ns, *P* > 0.05; Fig. 3d). Collectively, these data suggest that homologous desensitization occurs for both ANP- and CNP-dependent signaling, whereas cross-desensitization of GC-A and GC-B signaling does not. This further suggests that these ligands are exerting their effects via their specific cognate receptors in GH3 cells.

### CNP-induced down-regulation of Npr2 mRNA or GC-B protein does not occur in GH3 cells

**Fig. 4:**
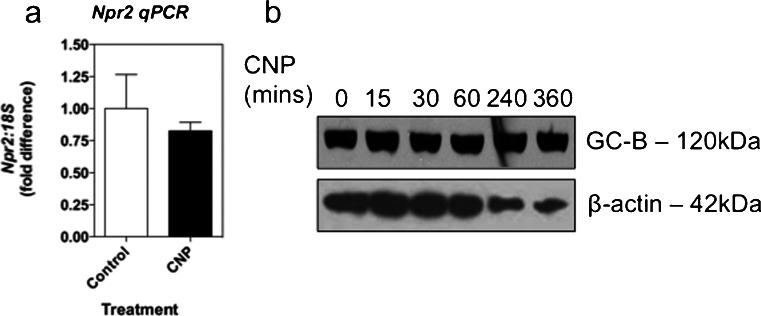
CNP fails to alter expression of *Npr2* mRNA or GC-B protein in GH3 somatolactotrope cells. **a** GH3 cells were stimulated with 0 or 100 nM CNP for 24 h prior to extraction of total RNA, cDNA synthesis, and quantitative PCR for endogenous *Npr2* expression. The data shown are means ± SEM of three independent experiments (*n* = 3). **b** GH3 cells were stimulated with 100 nM CNP for the indicated time, prior to extraction of total protein and Western blot analysis for GC-B expression. Blots were stripped and re-probed for β-actin. Panels are representative of three independent experiments (*n* = 3)

We next examined whether *Npr2* or GC-B down-regulation (as determined by a reduction in *Npr2* mRNA expression and or GC-B protein expression) mediated the inhibitory effects of sustained CNP exposure. The effect of CNP exposure on the expression of the endogenous *Npr2* gene was examined by using qPCR. A 24-h treatment with 100 nM CNP failed to alter *Npr2* expression in GH3 cells (Fig. 4a). To establish whether down-regulation occurred at the protein level, total proteins were extracted from GH3 cells incubated for up to 24 h with 100 nM CNP. Western blotting for GC-B protein revealed that CNP failed to alter expression levels (Fig. 4b). Collectively, these data suggest that a loss of *Npr2* mRNA or GC-B protein is not responsible for the reduced cGMP accumulation seen in pre-treated GH3 cells.

### Agonist-induced internalization does not accompany GC-A or GC-B desensitization

**Fig. 5:**
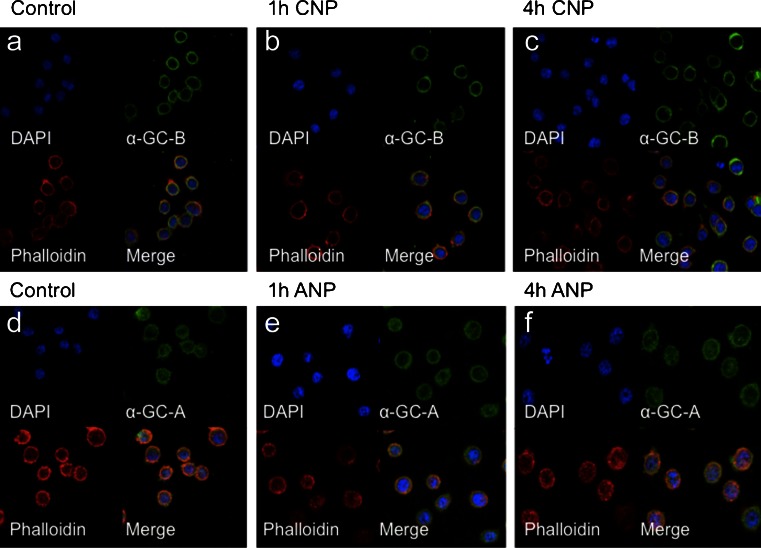
Lack of ligand-dependent internalization of GC-B or GC-A in GH3 somatolactotrope cells. GH3 cells were cultured on glass coverslips for 24 h prior to being treated with either 100 nM CNP for 0 (**a**), 1 h (**b**), or 4 h (**c**), or 100 nM ANP for 0 (**d**), 1 h (**e**), or 4 h (**f**). GC-A and GC-B immunoreactivity was detected by using Alexa488-conjugated secondary antibodies (*green*), whereas Alexa635-conjugated phalloidin was used to detect the actin cytoskeleton (*red*). Nuclear localization was detected by using 4,6-diamidino-2-phenylindole (*DAPI*) stain for orientation (*blue*). Panels are representative of three independent experiments (*n* = 3). Magnification ×40

To determine whether the GC-A or GC-B receptors undergo internalization upon stimulation with their respective ligands, confocal microscopy for GC-A and GC-B was used to visualize this process directly. Representative immunofluorescence for GC-A- and GC-B-labeled GH3 cells are shown (Fig. 5a–f). GH3 cells treated with 100 nM CNP or 100 nM ANP for 0–4 h did not demonstrate any observable GC-B or GC-A internalization, as observed by the overlay of the staining for actin filaments with that for GC-B and GC-A.

### Heterologous desensitization of GC-B signaling involves sphingolipid and calcium signaling in GH3 cells

**Fig. 6:**
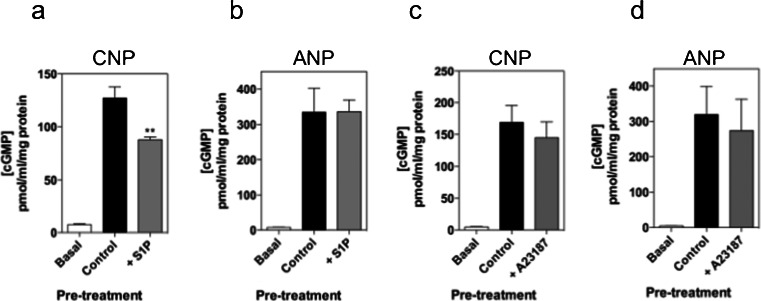
Evidence of sphingosine-mediated but not calcium-mediated, heterologous desensitization of GC-B signaling in GH3 somatolactotrope cells. GH3 cells were pre-treated with PSS containing either (**a**, **b**) 10 μM sphingosine-1-phosphate (*S1P*) or (**c**, **d**) 1 μM A23187 (calcium ionophore) for 30 min in the absence of IBMX, before a 15-min stimulation with either 100 nM CNP (**a**, **c**) or 100 nM ANP (**b**, **d**) in the presence of 1 mM IBMX. The data are means ± SEM of three independent experiments, each performed in triplicate and are expressed as pmol/mg protein (*n* = 3). **P < 0.01, significantly different from control response to CNP

Sphingolipids and calcium have been implicated in regulating natriuretic peptide signaling (Abbey-Hosch et al. 2004; Abbey and Potter 2002). Therefore, we examined the role of sphingosine-1-phosphate (S1P) on CNP-stimulated cGMP accumulation. Pre-treating GH3 cells with 10 μM S1P for 30 min failed to significantly alter basal cGMP accumulation (Fig. 6a). However, S1P pre-treatment did cause desensitization of CNP-stimulated cGMP accumulation (by 30 ± 2.7 % compared with control, **P < 0.01). Interestingly, pre-treating GH3 cells with 10 μM S1P did not result in desensitization of ANP-stimulated cGMP accumulation, compared with the ANP control samples (Fig. 6b; ns, *P* > 0.05), suggesting that GC-B and GC-A receptors are differentially regulated in GH3 cells. In similar experiments, cells were pre-treated with either 0 or 10 μM A23187 for 30 min prior to stimulation with either 0, 100 nM CNP, or 100 nM ANP for 15 min; however, pre-treatment with this calcium ionophore failed to alter either CNP- or ANP-stimulated cGMP accumulation (Fig. 6c, d).

### Heterologous desensitization of GC-B signaling involves PKC but not cAMP signaling in GH3 cells

**Fig. 7:**
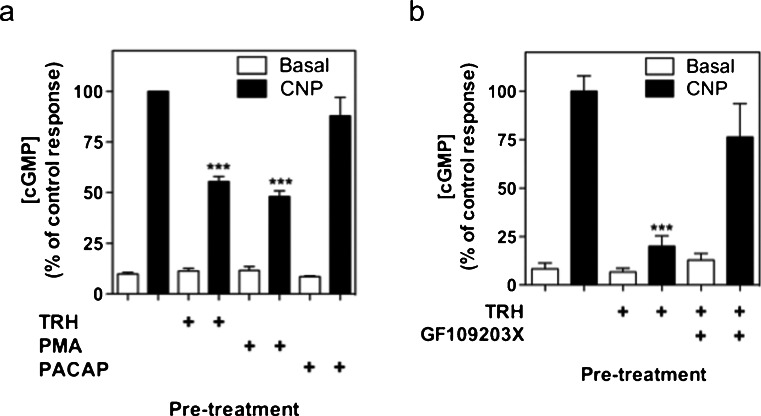
Protein kinase C (PKC)-dependent heterologous desensitization mediates the effects of thyrotrophin-releasing hormone (*TRH*) on CNP-stimulated GC-B signaling in GH3 somatolactotrope cells. **a** GH3 cells were pre-treated with PSS containing either 0 or 100 nM TRH, phorbol-12-myristate-13-acetate (*PMA*), or pituitary adenylate cyclase-activating polypeptide (*PACAP*) for 30 min in the absence of IBMX, before a 15-min stimulation with either 0 or 100 nM CNP in the presence of 1 mM IBMX. The data are means ± SEM of three independent experiments, each performed in triplicate and are expressed as % control reponse to CNP (*n* = 3). ***P < 0.001, significantly different from control response to CNP. **b** GH3 cells were pre-treated with PSS containing either 0 or 1 μM GF109203X for 30 min and a subsequent pre-treatment with 0 or 100 nM TRH in the continued absence or presence of GF109203X, before a 15-min stimulation with either 0 or 100 nM CNP in the presence of 1 mM IBMX. The data are means ± SEM of three independent experiments, each performed in triplicate and are expressed as % control reponse to CNP (*n* = 3). ***P < 0.001, significantly different from control response to CNP

Subsequent experiments examined the potential regulation of GC-B signaling by other peptide hormones. To determine whether the GC-B receptor underwent heterologous desensitization in GH3 cells, the effect of three known activators of PKC on CNP-stimulated cGMP accumulation was analyzed. Pre-treatment with TRH, with the phorbol ester, phorbol-12-myristate-13-acetate (PMA), or with pituitary adenylate cyclase-activating polypeptide (PACAP) failed to alter basal cGMP accumulation (ns, *P* > 0.05). However, cells pre-treated with 100 nM TRH or PMA showed a significant reduction in CNP-stimulated cGMP accumulation compared with the control (44.6 ± 2.5 % and 51.9 ± 2.8 %, respectively, ***P < 0.001). Pre-treating the GH3 cells with 100 nM PACAP failed to alter CNP-stimulated cGMP accumulation (ns, *P* > 0.05; Fig. 7a). To establish whether the TRH-induced heterologous desensitization of GC-B signaling was PKC-dependent, GH3 cells were pre-treated with either 0 or 1 μM GF109203X for 30 min to inhibit PKC activity. After this, cells were further pre-treated in the absence or presence of GF109203X and 0 or 100 nM TRH for a further 30 min, prior to stimulation with 0 or 100 nM CNP for a final 15-min stimulation in the presence of 1 mM IBMX. As shown (Fig. 7b), TRH pre-treatment caused the expected heterologous desensitization in GC-B signaling (to 20.1 ± 5.4 %, *P* < 0.001 compared with control) but this desensitization was predominantly reversed in the presence of GF109203X (to 76.4 ± 17.2 %, ns compared with control). Collectively, these data suggest that TRH, via the activation of PKC, causes heterologous desensitization of GC-B in GH3 cells.

## Discussion

Early expression profiling of CNP established the anterior pituitary as being a major site of expression and action (McArdle et al. 1993; Thompson et al. 2009; Fowkes et al. 2000; Sudoh et al. 1990; Komatsu et al. 1991). Whereas our previous studies have focused on the natriuretic peptide system of gonadotrope-lineage cells (McArdle et al. 1993; Thompson et al. 2009; Fowkes et al. 1999, 2000), data from mouse models of disrupted CNP or GC-B have indicated a potential deficiency in growth hormone secretion (Chusho et al. 2001; Tamura et al. 2004), raising the intriguing possibility that CNP/GC-B are involved in regulating somatotrope function. In the current study, expression profiling in somatolactotrope-derived GH3 cells has indicated the presence of both GC-A and GC-B receptors, which exhibit potent activation by either ANP or CNP in terms of cGMP accumulation, with similar EC_50_ properties. Despite these similar pharmacological properties, GH3 cells probably express more GC-A receptors, as the maximal response to ANP is almost twice that of CNP. However, our previous studies of primary cultures of rat pituitary cells indicated a more comparable effect of these peptides on cGMP accumulation (Thompson et al. 2009), potentially reflecting differences between primary and immortalized cells but also the relative ratio of GC-A and GC-B expression in the different cell types.

The desensitization of natriuretic peptide receptors has been extensively studied in cell lines stably transfected with GC-A or GC-B (Potter and Hunter 1998a, 1998b; Potter and Garbers 1994) and typically involves the dephosphorylation of key Ser and Thr residues (Potter 1998). However, studies reporting the desensitization of endogenous GC-A and GC-B are limited (Fowkes et al. 2000; Müller et al. 2006). Our current data represent the first demonstration that the desensitization of endogenous GC-A and GC-B signaling occurs in GH3 somatotrope cells.

Experimental paradigms to investigate receptor desensitization are often performed in one of two ways: time-course studies of a single continuous exposure to ligand or pre-treatment and subsequent re-exposure to the same ligand. Our current studies involved the use of both of these approaches to examine particulate guanylyl cyclase signaling in GH3 cells, similar to previous studies investigating PACAP signaling in gonadotropes (McArdle and Forrest-Owen 1997). Time-course studies of CNP-stimulated cGMP accumulation in GH3 cells have shown a rapid elevation of cGMP levels within 5 min of CNP treatment, reflecting the activation of GC-B receptors. Importantly, the continued accumulation of cGMP between 5 to 15 min of CNP treatment does not significantly increase, suggesting that CNP-stimulated cGMP accumulation in GH3 cells undergoes rapid homologous desensitization. This is in agreement with the effects of CNP on GC-B signaling in αT3-1 cells (Fowkes et al. 2000) and suggests that potential autocrine or paracrine effects of CNP in both somatotrope/lactotrope and gonadotrope lineage cells can be regulated by homologous desensitization. Interestingly, the same rapid homologous desensitization is not observed in the presence of ANP suggesting that the mechanisms of GC-A and GC-B desensitization differ in GH3 cells. One potential explanation for the lack of rapid homologous desensitization of ANP signaling in GH3 cells might be the apparent abundance of GC-A receptors compared with GC-B receptors. Certainly, the qRT-PCR *Npr1* expression data, coupled with the potent concentration-response curve of ANP-stimulated cGMP accumulation in GH3 cells, support this interpretation. As the receptor number is known to influence receptor desensitization (Rousseau et al. 1997), ANP-stimulated cGMP accumulation might not result in rapid homologous desensitization of GC-A receptors within the short 15-min time-course in the current studies.

Dephosphorylation of exogenously expressed GC-A or GC-B receptors has long been established as a major mechanism by which natriuretic peptide signaling is regulated (Potter et al. 2006; Potter and Garbers 1992, 1994; Yoder et al. 2012). Therefore, in order to determine whether the rapid homologous desensitization of endogenous GC-B receptors in GH3 cells was also dependent upon dephosphorylation, we examined the effect of inhibiting protein phosphatase activity. The selective PP2A inhibitor, OA, partially reversed the failure to maintain the initial rate of cGMP accumulation. However, inhibition of PP2B activity with cypermethrin or protein tyrosine phosphatases with Na_3_VO_4_ failed to reverse the apparent homologous desensitization, suggesting that PP2A but not PP2B or protein tyrosine phosphatases, activity was required for the regulation of GC-B signaling in GH3 cells. Our novel observations, namely that GC-B receptors are potential targets of PP2A-mediated dephosphorylation, indicate potential new targets for manipulating natriuretic peptide signaling. Recent studies investigating endogenous GC-A receptors in MA-10 Leydig cells have shown that homologous desensitization of these receptors is partially mediated selectively through calcineurin/PP3 (Henesy et al. 2012). Thus, tissue-specific phosphatase-specific control of guanylyl cyclase desensitization probably represents an emerging layer of complexity in the control of these receptors.

To extend our investigations of GC-A and GC-B signaling in GH3 cells, we used an alternative treatment paradigm to establish whether pre-exposure to ligand could cause desensitization to subsequent stimulation with either ANP or CNP. By using this approach, both GC-A and GC-B receptors appeared to undergo homologous desensitization in GH3 cells. Therefore, in addition to undergoing rapid homologous desensitization, the GC-B receptor in GH3 cells can be regulated by CNP over a prolonged period of time. Although the GC-A receptors appear to undergo homologous desensitization following prolonged exposure to ANP, this effect is not significant until at least 30 min of ANP pre-treatment. This discrepancy between the rates of onset of desensitization between GC-B and GC-A can be explained either by a distinct mechanism of desensitization compared with GC-B receptors or as being attributable to a difference in GC-A receptor number, as mentioned previously. Rapid homologous desensitization of endogenous GC-A receptors has been reported in MA-10 Leydig cells (Müller et al. 2006) suggesting that GC-A regulation may well be cell-type specific and thus warrants further investigation to establish the mechanism(s) involved.

As activation of either GC-B or GC-A signaling leads to an increase in cGMP concentrations, we examined whether stimulation of each receptor would lead to the desensitization of the other receptor. Both ANP and CNP can activate the reciprocal receptor, albeit only at pharmacological concentrations (Suga et al. 1992). Our demonstration of desensitization of GC-B and GC-A receptors in GH3 cells might not be entirely attributable to the consequence of elevated cGMP signaling, since neither ANP or CNP pre-treatment cause subsequent desensitization of their reciprocal receptors, although this might reflect the importance of receptor-specific sub-cellular compartmentalized cGMP production. These findings are in agreement with those observed in αT3-1 cells (Fowkes et al. 2000) in which ANP and CNP fail to desensitize the other receptor.

Prolonged exposure of GC-B receptors to CNP in GH3 cells might result in receptor down-regulation and internalization. In the current study, we failed to see any significant effect of prolonged exposure to CNP on endogenous *Npr2* mRNA or protein levels. Furthermore, GC-B immunoreactivity in GH3 cells co-localized with a marker of the cytoskeleton and was unaltered following CNP treatment. These observations suggest that rapid alterations in either the expression or localization of the GC-B receptor do not occur in GH3 cells. This lack of ligand-induced receptor internalization is in agreement with previous studies in 293 T cells (Fan et al. 2005). Internalization of GC-A and GC-B remains a controversial issue, as a number of studies have shown GC-A internalization in PC-12 cells and MA-10 cells (Rathinavelu and Isom 1991; Pandey 1993). However, several other studies have failed to demonstrate such internalization (Koh et al. 1992; Vieira et al. 2001) raising the possibility that the internalization of guanylyl cyclase receptors is a cell-type-specific phenomenon.

Heterologous desensitization of the natriuretic peptide receptors has been widely reported. Sphinogosine signaling is implicated in GC-B desensitization in NIH3T3 fibroblasts, A10 vascular smooth muscle cells and 293 T cells (Abbey-Hosch et al. 2004, 2005). These studies have revealed that the mechanism responsible for the S1P desensitization does not require PKC activation but is reliant on the elevation of intracellular Ca^2+^ levels, with ionomycin having a similar effect. In the current study, we observed a modest effect of S1P pre-treatment of CNP-stimulated cGMP accumulation in GH3 cells but ANP-stimulated cGMP accumulation is unaffected. Furthermore, the calcium ionophore A23187 has no effect on either GC-B or GC-A signaling, suggesting that particulate guanylyl cyclase signaling in GH3 cells is less sensitive to changes to intracellular calcium levels, as is the situation in gonadotrope-derived αT3-1 cells (McArdle et al. 1993).

The other major mechanism mediating heterologous desensitization of GC-A and GC-B signaling is the PKC pathway (Potter et al. 2006). We examined the effects of pre-treatment with TRH, PMA, or PACAP. Pre-treatment with PACAP fails to alter GC-B activity suggesting that limited activation of PKC occurs in response to PACAP in GH3 cells. This is in agreement with the predominant expression of vasoactive intestinal peptide activated receptor 2, which is known preferentially to activate cAMP and PKA (Hao et al. 2006). In contrast, pre-treatments with two known activators of PKC, TRH and PMA do cause a significant reduction in GC-B activity. PMA has previously been shown to desensitize GC-B activity in 293 T cells and αT3-1 cells (Fowkes et al. 2000; Abbey-Hosch et al. 2005). The novel observation that TRH can cause the heterologous desensitization of GC-B signaling in GH3 cells is at least partially dependent upon PKC activity, because GF109203X is capable of reversing the effect, as has been described previously in human airway smooth muscle cells (Hamad and Knox 1999). We have previously shown that another known activator of PKC, namely gonadotrophin-releasing hormone, can cause the heterologous desensitization of GC-B signaling in αT3-1 cells (McArdle et al. 1994; Fowkes et al. 2000). PKC-dependent heterologous desensitization of GC-B is thought to increase the activity of a protein phosphatase that specifically dephosphorylates Ser^523^ (Potter and Hunter 2000). Establishing the identity of this phosphatase will reveal a potential therapeutic target by which to regulate guanylyl cyclase signaling.

Both humans and mice with mutations in the genes encoding either CNP or its receptor, GC-B, suffer from severe growth disorders (Chusho et al. 2001) and, in some cases, growth hormone deficiency (Tamura et al. 2004). Despite significant homology between the three natriuretic peptides and between their receptors, mutations in the genes encoding ANP, BNP, or GC-A fail to phenocopy CNP/GC-B-disrupted mice (Kishimoto et al. 2011). Our recent observations of GC-B expression in normal fetal pituitary tissue and in a range of human pituitary adenomas (including those from acromegalics) strongly support a pituitary role for CNP and GC-B signaling, particularly within somatotrope-lineage cells (Thompson et al. 2012). Our current findings that endogenous GC-B receptors in GH3 cells are sensitive to both homologous and heterologous desensitization, without apparent down-regulation or internalization, provide an opportunity to manipulate GC-B/cGMP signaling in somatotropes. Given that both CNP and cGMP have previously been shown to act as growth hormone secretogogs (Hartt et al. 1995; Shimekake et al. 1994), we are tempted to speculate that such manipulation of guanylyl cyclase signaling might offer potential therapies for growth-related disorders.
